# Sustained Circulating Bacterial Deoxyribonucleic Acid Is Associated With Complicated *Staphylococcus aureus* Bacteremia

**DOI:** 10.1093/ofid/ofz090

**Published:** 2019-02-26

**Authors:** Johnny Gutierrez, Alessander O Guimaraes, Nicholas Lewin-Koh, Aklile Berhanu, Min Xu, Yi Cao, Janice Kim, Donghong Yan, Joanna K Chang, Jason B Dinoso, Catherine A Koss, Angelo Clemenzi-Allen, Henry F Chambers, Melicent C Peck, Amos Baruch, Carrie M Rosenberger

**Affiliations:** 1 Genentech, Inc., South San Francisco, California; 2 University of California San Francisco, California

**Keywords:** bacteremia, cell-free DNA, circulating DNA, prognostic biomarkers, *Staphylococcus aureus*

## Abstract

**Background:**

*Staphylococcus aureus* (SA) bacteremia often requires a long treatment duration with antibiotics to prevent relapse due to the ability of SA to establish reservoirs of infection in sites such as heart and bone. These metastatic sites of infection cannot be serially sampled to monitor the clearance of SA infection. This study aimed to establish a link between persistence of circulating SA deoxyribonucleic acid (SA-DNA) and tissue reservoirs in patients with SA bacteremia.

**Methods:**

A highly sensitive quantitative polymerase chain reaction was used to measure whole blood SA-DNA and plasma-derived SA cell-free DNA (SA-cfDNA) in a set of longitudinal samples from 73 patients with confirmed SA bacteremia and correlated with clinical features.

**Results:**

Blood SA-DNA was detected for longer than the duration of positive blood cultures. Longer duration of circulating bacterial DNA was observed in complicated SA bacteremia infections, such as endocarditis and osteoarticular infections, compared with uncomplicated bloodstream infections. In contrast, traditional blood cultures demonstrated similar time to clearance regardless of foci of infection. Plasma-derived SA-cfDNA showed concordance with blood SA-DNA levels. Baseline levels of SA-DNA were higher in patients presenting with greater clinical severity and complicated bacteremia.

**Conclusions:**

Prolonged levels of circulating SA-DNA in patients with complicated tissue reservoirs after clearance of blood cultures observed in this single-center study should be validated in additional cohorts to assess the potential utility for monitoring clearance of infection in patients with SA bacteremia.

*Staphylococcus aureus* (SA) bacteremia is a major health threat and is responsible for many serious blood, tissue, and device-related infections [1]. Even in the presence of susceptible standard-of-care (SOC) antibiotic, SA is difficult to treat due to metastatic reservoirs of infection, such as heart and bone [2–5]. *Staphylococcus aureus* that have spread to tissues require a long treatment duration with antibiotics for clearance to prevent relapse [6], and these tissue reservoirs cannot be serially sampled during the treatment course to monitor clearance of infection.

Blood culture (BC) remains the gold standard to diagnose bloodstream infections, and persistently positive BCs are one of the strongest predictors of morbidity and mortality [7]. However, negative BCs do not indicate clearance of tissue foci of infection, with the majority of BCs turning negative within 5 days of starting appropriate antistaphylococcal therapy even in cases of SA infections such as endocarditis that typically require 6 weeks or more of antibiotic therapy [6]. Hence, biomarkers that would enable rapid identification of patients with persistent reservoirs of infection or serve as an early marker of relapsing infection would be of high clinical value to guide antibiotic treatment decisions, including the development of novel therapies.

Utilizing molecular methods to monitor bacterial load offers several advantages over BC because they are unaffected by antibiotic treatment and deliver a quantitative same-day result using lower sample volume than traditional BC [8, 9]. Therefore, bacterial polymerase chain reaction (PCR) assays have been developed to assess bacterial load and evaluate the potential relationship between blood bacterial load and disease severity [10–12]. Studies have also shown that elevated levels of cell-free human deoxyribonucleic acid (cfDNA), released from necrotic and apoptotic cells, is a predictor of mortality in bacteremia and sepsis [13–15]. We hypothesized that death of SA-infected cells in response to bactericidal antibiotics and the immune response could release *Staphylococcal* cfDNA and offer a biomarker of disease that can be measured directly in serum or plasma.

In this study, we describe the development of a highly sensitive method to quantify circulating SA-DNA directly in whole blood, as well as SA-cfDNA from plasma. These assays were used to quantitate levels of SA-DNA in longitudinal samples from patients with SA bacteremia. We examined relationships between SA-DNA quantified in blood with infection foci and clinical metrics. In patients with complicated SA bacteremia, detection of bacterial DNA in blood was sustained after clearance of BCs.

## METHODS

### Clinical Samples

The study was performed in collaboration with the University of California, San Francisco, at San Francisco General Hospital under a protocol approved by the Institutional Ethics Review Board. Patients with BCs positive for SA were included in this study. All subjects received appropriate SOC antistaphylococcal antibiotic therapy per the treating physician. All blood and serum assays were performed on convenience samples collected retrospectively from leftover whole blood and plasma drawn for routine clinical laboratory measurements. Samples were collected from 73 unique patients for analysis.

The following clinical data (when available) were collected from the medical record: sex, age, vital signs (systolic and diastolic blood pressure, pulse rate, respiration, body temperature), comorbidities, source of infection, time-to-positivity and time to clearance of BC, and timing of administration of antibiotics in relation to BC collection. A negative BC was defined as no growth at 5 days of culture. Clinical severity was defined by the site study physician (A.C.-A. or C.A.K.) as septic shock (organ dysfunction) or severe sepsis (hypotension persisting despite adequate fluid resuscitation). Complicated SA bacteremia is defined as patients for whom the treating infectious disease physician recommended >2 weeks of SOC antistaphylococcal antibiotics or who died. Routine cultures were performed on ~10-mL blood samples. Microbiological failure was defined as a BC positive for SA for ≥5 days on appropriate antibiotic therapy. Prolonged treatment duration was defined as length of prescribed antibiotic therapy >1 month (31+ days). A sustained elevation in white blood cell (WBC) count was defined as >10K cells/μL for >10 days. Mortality was measured as in-hospital mortality. Five patients died during their hospitalization. One death due to septic shock was attributed to SA infection; the other 4 deaths were not deemed attributable to SA. The relatively low mortality rate (6.8%) may reflect not enrolling patients who died early due to lack of sample availability, this not being a tertiary referral hospital, patient population, and the use of in-hospital mortality rather than 30-day mortality due to the inability to follow-up with patients.

### Procalcitonin Assay

Procalcitonin (PCT) concentrations were measured in serum samples using the ADVIA Centaur BRAHMS PCT assay. The functional sensitivity was 0.05 ng/mL, but, to conserve sample volume, some patient samples were prediluted in specified multidiluent and have lower limit of detection of 0.2 ng/mL.

### Mouse Infection Model

All in vivo studies were approved by the Institutional Animal Care and Use Committee at Genentech, Inc. and were conducted in compliance with the regulations of the Association for Assessment and Accreditation of Laboratory Animal Care. The SA acute infection mouse model was described previously [5]. A/J mice were infected with SA by administration of USA300 strain (NRS384; BEI Resources) at 2 × 10^6^ colony-forming units (CFU) per mouse via intravenous tail injection. Blood and kidney samples were collected from 3 mice each at 10 minutes, 6, 24, and 48 hours postinfection. For the persistent infection model, C57BL/6 or Balb/c (Charles River/Hollister) mice were infected intravenously with 1–2 × 10^7^ CFU USA300, and femur and tibia bones were collected from 10 mice each after 3, 26, 35, or 60 days after infection. In this model [16], bacteria seed the tissues from the bloodstream, leading to persistent bone infection for >60 days, with histological features of human osteomyelitis, along with metastatic seeding to organs such as kidney.

### Colony-Forming Unit Measurements

Kidneys and blood were collected from each mouse after euthanasia at described time points above and CFU counts were determined. See Supplementary Data for details.

### Isolation of Bacterial Deoxyribonucleic Acid

For mouse studies, kidney (250 μL, 1/20th of homogenate) and bone homogenates (200 μL, 1/10th of homogenate) were treated with bacteria lysis buffer (Roche, catalog no. 04659180001) supplemented with lysostaphin (Sigma, catalog no. L4402) and lysozyme (Sigma, catalog no. L1667) and incubated at 37°C for 10 minutes, followed by Proteinase K (Roche, catalog no. 03115887001) treatment at 55°C for an additional 10 minutes. The suspension was added to tubes with garnet beads and subjected to mechanical lysis (Precellys) for 1 minute. Samples were centrifuged at 16 000 ×*g* for 1 minute, and lysates were transferred to MagNA Pure Compact (Roche) sample tubes for automated DNA extraction per manufacturer instructions.

For patient samples, total bacteria-associated staphylococcal DNA was measured in whole blood, and cell-free staphylococcal DNA was measured in plasma (see Supplementary Figure 1). A total of 500 μL of patient whole blood was treated with red blood cell lysis solution (Roche) at room temperature for 10 minutes and then centrifuged at 16 000 ×*g* for 5 minutes to pellet and enrich bacteria. After discarding the supernatant, the pellets were resuspended in bacteria lysis buffer and subjected to the procedure described above for bacterial nucleic acid isolation. For cfDNA isolation, to remove intact bacteria, 150 μL of patient plasma was centrifuged at 16 000 ×*g* for 5 minutes, and, without disturbing the pellet, the top 100 μL was transferred to MagNA Pure Compact (Roche) sample tubes for automated DNA extraction.

### Primers and Probes

For group-specific detection of *Staphylococcus*, custom primers and TaqMan probes were designed to an alignment of 16S ribosomal ribonucleic acid (rRNA) gene region specific for *Staphylococcus*: 16S-01-FP (5’-CCGCAT GGTTCAAAAGTGAAA-3’), 16S-01-RP (5’-GCAGCGCGG ATCCATCTA T-3’), 16S-R-FAM-NFQ (ACGGTCTTGCTG TCACT). Because these primers detect all species of *Staphylococcus*, only samples from patients with culture-confirmed monomicrobial SA bacteremia were included in this study. The specificity of the TaqMan probes was tested against SA strains and other prevalent bacterial species known to cause bacteremia (Supplementary Table 1).

### Quantitative Polymerase Chain Reaction

Detailed procedures can be found in Supplementary Data. In brief, quantitative PCR (qPCR) was carried out on the purified sample DNA and known copy number of DNA standard. The 16S rRNA gene copy number can vary from 5 to 6 copies per bacterium so measurements reflect total genome copies rather than an absolute measurement of bacterial load.

### Statistical Analysis

Because timing of BC collection was based on physician preference, it was only possible to define the interval between the last positive BC and the first negative BC. The majority of patients were interval censored, and the interval coded as [Time of last positive sample, Time of 1st negative sample]. A smaller number of patients were left or right censored: if there was no positive BC after the positive index culture, the interval for clearing bacteremia was considered left censored and coded as (-Infinity, Time of 1st negative sample]. If no negative BC was collected, the time of the first negative sample was considered right censored and the duration of bacteremia coded as [Time of last positive sample, Infinity). All patients who died (n = 5) were considered right censored if they did not clear bacteremia before death. Although we acknowledge that death is a competing risk, because our analysis was focused on comparing time to clearance of SA using BC versus molecular methods, patients who died were considered right censored. Time to event curves for interval-censored data were estimated using the nonparametric maximum likelihood estimate (NPMLE), which provides a generalized survival distribution similar to a Kaplan-Meier curve for interval-censored data [17, 18]. Median time to clearance and confidence intervals (CIs) were estimated from the NPMLE by linear extrapolation from the upper and lower ends of the intervals bracketing the median. Estimation of the NPMLE was done using the interval package [18]. All analysis was done using the statistical computing language R (R Core Team 2018, version 3.5.1).

## RESULTS

### Correlation of Colony-Forming Units With *Staphylococcus aureus* Deoxyribonucleic Acid in Mouse Infection Models

**Figure 1.**
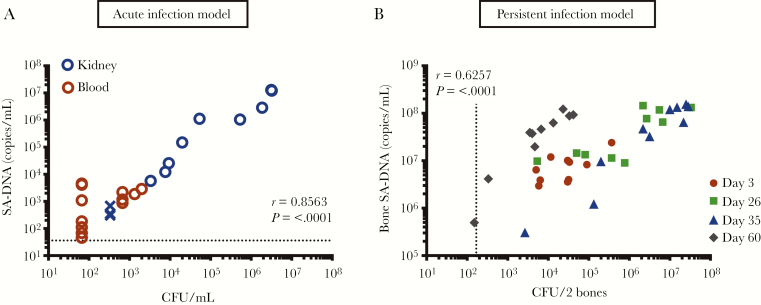
(A) Correlation of agar culture colony-forming unit (CFU) count with *Staphylococcus aureus* deoxyribonucleic acid (SA-DNA) quantitative polymerase chain reaction (qPCR) copies/mL in acute and persistent infection mouse models. Spearman correlation of culture CFU with relative qPCR counts in kidneys and blood samples from mouse acute infection model and (B) bone samples from persistent mouse infection model. Each symbol represents the counts from an individual infected animal. X symbols indicate no colonies were detected, and all samples were detected by qPCR. Limit of detection 333 CFU/kidney, 167 CFU/2 bones, 67 CFU/mL for blood, and 36 copies/mL for SA-DNA qPCR assay.

We used acute and persistent SA mouse infection models to determine how SA-DNA qPCR levels correlate with CFU [16]. In the acute infection model, SA-DNA and CFU from blood and kidney samples taken up to 48 hours postinfection were strongly correlated in samples above the detection limit (Spearman rho = 0.8563, *P* ≤ .0001) (Figure 1A). *Staphylococcus aureus* DNA was detected in all blood and kidney samples, whereas CFU were below the detection limit (67 CFU/mL) in 7 of 12 blood samples. In the persistent infection model, mice develop osteomyelitis with a high burden of CFU that persists for >60 days. Colony-forming units and qPCR SA-DNA copies/mL from the SA isolated from the femur and tibia bones were significantly correlated and were detected in all samples up to 60 days postinfection (Spearman rho = 0.6257, *P* < .0001) (Figure 1B).

### Assessment of Circulating *Staphylococcus aureus* Deoxyribonucleic Acid Levels in Patients With *S aureus* Bacteremia

Longitudinal whole blood and plasma samples were convenience sampled from 73 patients with confirmed SA bacteremia by BC while hospitalized (up to 48 days) (Supplementary Table 2). The median duration of antibiotic treatment at the time of first sample collection was 2 days. All baseline analyses were performed in samples collected within 3 days from the initiation of appropriate antibiotic therapy for SA.

**Figure 2.**
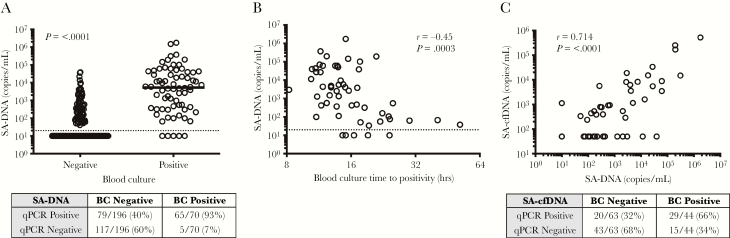
Quantitative polymerase chain reaction (qPCR) bacterial loads in bacteremic patients. (A) Blood bacterial qPCR counts in samples collected at baseline and after antibiotic treatment, in relation to whether corresponding blood culture (BC) at the time of qPCR testing was positive versus negative. Medians and Mann-Whitney are shown, and the table indicates the number of samples in which deoxyribonucleic acid (DNA) was detected (positive) or undetectable (negative) in the qPCR assay. All subjects had positive index BCs. (B) Spearman correlation of baseline blood qPCR bacterial load with BC time to positivity. Limit of detection (20 copies/mL; denoted by a dashed line). (C) Spearman correlation of plasma *Staphylococcus aureus* cell-free DNA (SA-cfDNA) and blood SA-DNA levels in bacteremia patients at initial collection.

Samples collected from patients with positive BC had significantly higher copy numbers of SA-DNA compared with samples collected from patients with negative BC at the time of sampling for PCR (Mann-Whitney, *P* ≤ .0001) (Figure 2A). *Staphylococcus aureus* DNA was detected in 93% of the blood samples that were BC positive and in 40% of the samples that were BC negative at the time of sampling for PCR. *Staphylococcus aureus* DNA counts correlated with initial BC time-to-positivity, which has been proposed as a semiquantitative measure of blood bacterial load (Spearman rho = −0.53, *P* < .0005) (Figure 2B) [19]. Seven percent of samples were positive by BC and negative by PCR (Figure 2A), which could result from the much larger blood volumes used for BC (~10 mL) than available for PCR (0.5 mL). There was no significant difference in initial SA-DNA levels between infections caused by methicillin-susceptible SA (MSSA) versus methicillin-resistant SA (MRSA) (MSSA median = 2030 copies/mL, ranges 10–374 130 and MRSA median = 557 copies/mL, range 10–1 760 726).

Because bactericidal activity from antibiotics and the action of the immune system may result in release of bacterial DNA, we sought to investigate whether SA-cfDNA was present in circulation and whether levels of SA-cfDNA and SA-DNA were correlated. *Staphylococcus aureus* cfDNA was detected in 66% of plasma samples from patients with positive BC compared with 32% of samples from patients with negative BC at the time of plasma sampling. *Staphylococcus aureus* cfDNA showed significant correlation to total blood SA-DNA (*P* < .0001, r = 0.7378) (Figure 2C). The sensitivity of the assays cannot be directly compared because DNA was extracted from 5 times more volume of blood than of plasma, due to sample availability.

**Figure 3.**
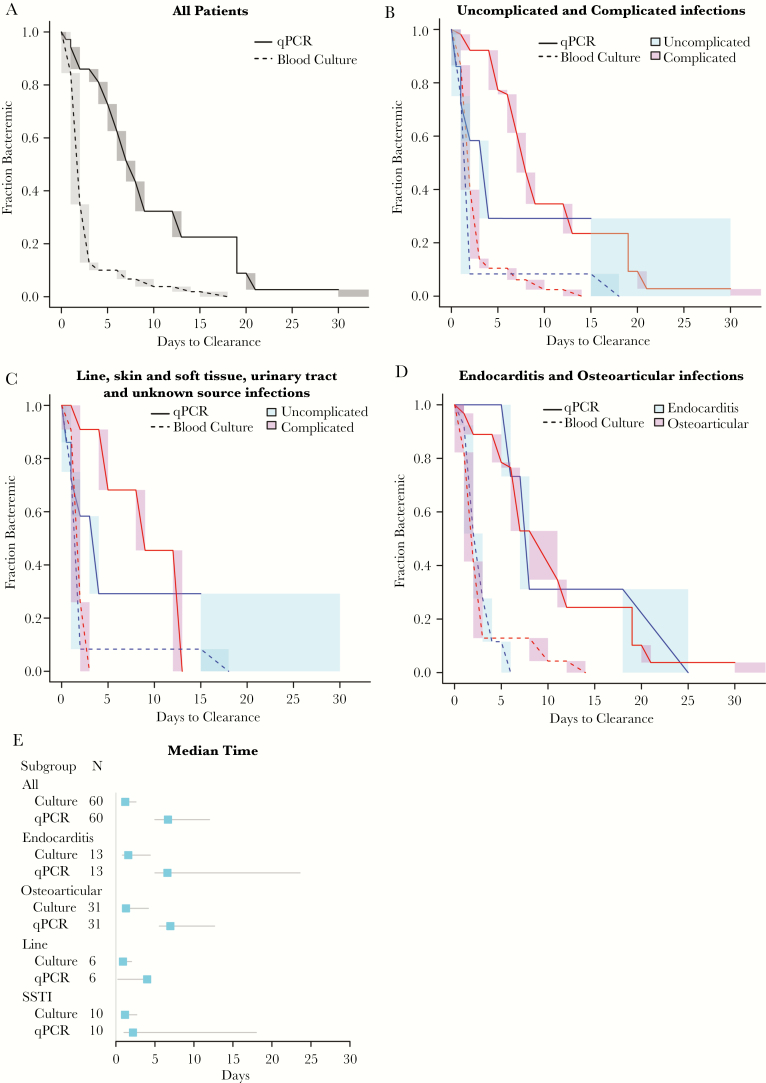
Comparison between the duration of circulating *Staphylococcus aureus* deoxyribonucleic acid (SA-DNA) levels and duration of blood culture (BC) positivity. (A) Time to clearance of *S aureus* by BC (dotted lines) and SA-DNA (solid lines) by quantitative polymerase chain reaction (qPCR) in all patients. (B) Time to clearance of *S aureus* by BC (dotted lines) and SA-DNA (solid lines) in uncomplicated versus complicated infections. (C) Time to clearance of *S aureus* by BC and SA-DNA of catheter, skin and soft tissue infection (SSTI), urinary tract infection, and unknown infection source categorized into uncomplicated and complicated infections. (D) Time to clearance of *S aureus* by BC and SA-DNA in osteoarticular and endocarditis infections. Shading indicates the uncertainty in the interval censored data. (E) Comparison of the median time to clearance of *S aureus* as measured by BC (1.3 days) and SA-DNA by qPCR (6.3 days) in all subjects and subdivided by source of infection. Lines represent a 95% confidence interval around the median.

*Staphylococcus aureus* DNA was detectable for a median of 6.8 days (95% CI, 5.3–12.6; range, 0–30 days) in blood samples collected after antibiotic treatment, which was considerably longer then the median duration of detection of SA by BC of 1.3 days (95% CI, 1.0–2.7; range, 1–15 days) (Figure 3A). Higher SA-DNA levels in the index sample correlated with a longer median time to SA-DNA clearance in serial samples (Supplementary Figure 2). Subjects with complicated SA bacteremia infections have a median time to clearance of <2 days by BC compared with 7.3 days by SA-DNA qPCR (Figure 3B). We observed a relationship between foci of infection and median time to clearance of SA-DNA (Figure 3E). The median time to clear SA-DNA in line infections was 5.9 days (95% CI, 3.2–19), and the median time to clearance of skin and soft tissue infections (SSTIs) was 3.2 days (95% CI, 0.8–18.0). When line and SSTI infections were subcategorized into complicated and uncomplicated infections, a longer median clearance of 7.7 days was observed in complicated infections compared with 2 days in uncomplicated infections of SA-DNA (Figure 3C). A similar long median time to clearance of SA-DNA was measured in other complicated SA bacteremia with osteoarticular or endocarditis infection foci (osteoarticular: 7.0 days, 95% CI = 5.5–11.8; endocarditis: 6.6 days, 95% CI = 5.0–23.6) (Figure 3D). *Staphylococcus aureus* DNA could be detected for longer in MSSA compared with MRSA infections (Supplementary Figure 4).

### Association of Bacterial Load With Clinical Metrics

**Figure 4.**
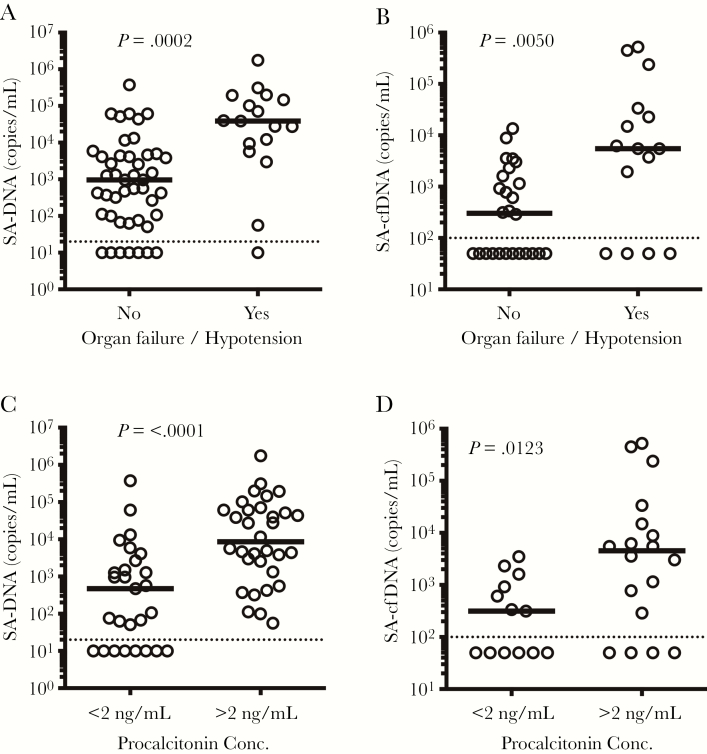
(A and B) Correlation of initial blood quantitative polymerase chain reaction bacterial load to clinical severity assessed at presentation. Initial *Staphylococcus aureus* deoxyribonucleic acid (SA-DNA) and SA cell-free DNA (cfDNA) levels are higher in patients presenting with organ dysfunction and/or hypotension persisting despite adequate fluid resuscitation. (C and D) Initial SA-DNA and SA-cfDNA levels are also higher in patients presenting with levels of procalcitonin >2 ng/mL, a protein marker correlated with sepsis severity. Medians and non-parametric Mann-Whitney *P* values are shown, and dashed line indicates assay limit of detection.

**Figure 5.**
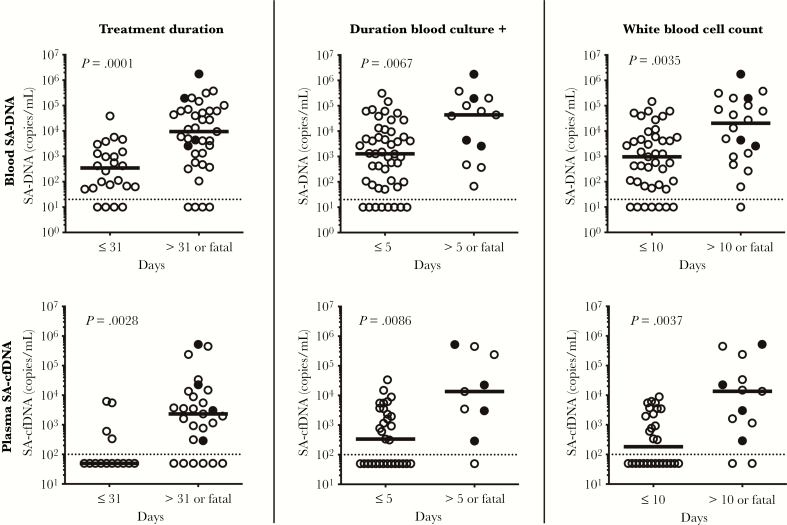
Associations between *Staphylococcus aureus* deoxyribonucleic acid (SA-DNA) levels and clinical metrics. (A) Antibiotic treatment duration >31 days or death, (B) time to negative blood culture >5 days or death, and (C) elevated white blood cell counts for >10 days or death. Filled circles indicate patients who died, medians and non-parametric Mann-Whitney *P* values are shown, and dashed line indicates lower limit of detection. cfDNA, cell-free DNA.

We also examined the relationship between circulating levels of SA-DNA and clinical metrics, hypothesizing that higher levels of SA-DNA may be associated with a more severe clinical course. Baseline levels of SA-DNA and SA-cfDNA were 64-fold and 18-fold higher, respectively, in patients presenting with organ dysfunction or hypotension compared with patients without these clinical features (Figure 4A and B). We also examined the relationship between SA-DNA and SA-cfDNA and serum PCT. Procalcitonin levels of >2 ng/mL have been shown to be associated with increased sepsis severity and mortality risk in critically ill patients [20–24]. *Staphylococcus aureus* DNA and SA-cfDNA were significantly higher in patients with PCT levels of >2 ng/mL (Figure 4C and D). Levels of SA-DNA and SA-cfDNA at presentation were significantly higher in patients with fatal outcome and requiring antibiotic treatment for >31 days, with prolonged bacteremia (BC positive >5 days), or with elevated WBC counts for >10 days (Figure 5). *Staphylococcus aureus* DNA and SA-cfDNA levels in the first available samples correlated with levels of blood neutrophils, but not total WBC count (Supplementary Table 3). Baseline level of SA-DNA was also correlated to median time to clearance of SA-DNA and BC (Supplementary Table 3). Taken together, higher baseline SA-DNA loads and a longer median duration of SA-DNA than BC positivity is observed in patients with complicated SA infections requiring longer courses of antibiotics.

## DISCUSSION

Treatment of complicated SA bacteremia requires a long course of antibiotics to prevent relapse. *Staphylococcus aureus* establishes reservoirs of infection in sites such as heart and bone that cannot be routinely or serially sampled to monitor clearance of SA bacteria. Blood culture is an insensitive tool to monitor clearance of these reservoirs. As a result, current treatment guidelines recommend prolonged antibiotic therapy after clearance of bacteria from the blood. In this study, we provide evidence that bacterial DNA as measured by qPCR persists in the circulation after traditional BC clearance in patients with SA bacteremia on appropriate antibiotic therapy. In addition, persistence of SA-DNA is correlated with deep foci of infection, such as osteomyelitis and endocarditis, found in patients with complicated bacteremia. The persistent levels of SA-DNA detectable after BC become negative in these patients suggests a potential way to monitor persistent foci of infection. An additional advantage of the qPCR assay is that it detects bacteria that do not grow in BCs from patients after antibiotic treatment.

We observed higher levels of SA-DNA in patients with longer time to clearance of BCs, deep tissue foci of infection, and persistently elevated WBC counts, suggesting a correlation between SA-DNA and measures of clinical severity. The qPCR assay for SA-DNA may be measuring live or dead bacteria in blood or phagocytes, and SA-cfDNA is measuring cfDNA released by killed bacteria. Our observations are in agreement with previous studies showing an association between high baseline bacterial DNA load by qPCR and greater severity (mortality or need for hospitalization) in patients with SA, *Mycoplasma pneumonia*, and *Neisseria meningitidis* infections [10–12, 25]. *Staphylococcus aureus* cfDNA levels measured in plasma were also higher in patients with greater clinical severity, but sparser longitudinal sampling and smaller available volumes for plasma prevented us from directly comparing between the 2 PCR assays. The strong correlation of SA-cfDNA with blood SA-DNA and the ease of cfDNA isolation compared with the more intricate isolation of intact SA-DNA merits measurement of SA-cfDNA in larger cohorts of patients with SA bacteremia.

## CONCLUSIONS

Future studies in larger prospective cohorts of patients with invasive SA infections are needed to address the limitations of this small pilot study. Uniformly sampled matched BC, blood DNA, and plasma DNA samples were not available for subjects in this study, which relied on convenience sampling. Larger volumes of matched samples are needed to assess the relative abundance of SA-DNA in whole blood versus serum versus SA-cfDNA in plasma, to ascertain which sample type will be the most sensitive for monitoring response to treatment. This would help to interpret whether the small proportion of samples that were positive by BC but negative by PCR results from differences in available blood volume (~10 mL BC, 0.5 mL blood PCR, 0.1 mL plasma PCR), differences in handling and storage of the convenience samples used in this study, or assay sensitivity. Evaluating the relationships between initial SA-DNA levels and metrics of clinical severity, such as mortality, relapse, or development of sepsis, are needed to extend the preliminary associations observed in this study. Because the current assay detects all species of *Staphylococcus*, this PCR approach must be paired with conventional microbiological diagnosis to exclude infection with other *Staphylococcus* strains as was done in this study. Future refinements of this assay could include multiplexing other genes to aid in increasing specificity. If the data from this pilot study is validated in larger cohorts, monitoring levels of SA-DNA during treatment may be informative as a marker of clinical resolution or relapse. Biomarkers that monitor clearance of tissue infection foci are essential to better inform the optimal duration of antibiotic treatment. This study provides preliminary evidence to support further evaluation of this rapid and sensitive SA quantitative PCR assay.

## Supplementary Data

Supplementary materials are available at *Open Forum Infectious Diseases* online. Consisting of data provided by the authors to benefit the reader, the posted materials are not copyedited and are the sole responsibility of the authors, so questions or comments should be addressed to the corresponding author.

Supplement-Information
