# Supplementary material for: Sustained Circulating Bacterial Deoxyribonucleic Acid Is Associated With Complicated Staphylococcus aureus Bacteremia
Source: Open Forum Infect Dis. 2019 Feb 26;6(4):ofz090. doi: 10.1093/ofid/ofz090 (PMC6475589; doi:10.1093/ofid/ofz090)
Supplement: Supplement-Information [file ofz090_suppl_supplement-information.pdf]

SUPPLEMENTARY INFORMATION

Supplemental Material and Methods

CFU measurements

Both kidneys and blood were collected from each mouse as described following end point euthanasia at selected time points. Both kidneys were homogenized in 5 mL PBS in M tubes using a gentleMACS^{TM} Dissociator (Miltenyi Biotec, Inc; Catalog # 130-093-235). Femurs and tibias were homogenized in 2 mL of PBS using 2.8 mm ceramic tubes (OMNI International; Catalog #19-628) for 2x30 second cycles in a Bead Ruptor homogenizer (OMNI International). Blood was treated with 0.1% Triton X-100 to lyse all blood cells. Ten-fold serial dilutions of tissue homogenates and treated blood were spread on blood agar plates (Teknova) and incubated under aerobic conditions at 37°C for 14-18 hours. The total bacterial burden in the kidneys and blood was determined by counting colonies in the highest dilution with non-overlapping colonies followed by multiplying the colony count with the dilution factor; the final product being expressed as CFU per organs. The detection limit was 133 cfu/bones (2 femur + 2 tibia), 400 cfu/kidneys, and 67 cfu/mL blood.

Quantitative PCR

Genomic DNA from *S. aureus* USA300 (FPR3757) was extracted and used as the standard. The mass of the genomic DNA and concentration was used to calculate 16S copy number for the standard curve. Quantitative PCR was carried out on the purified sample DNA and known copy number of DNA standard. Briefly, 20 µl reaction mixtures

containing 5 µl DNA, 10 µl 2x Taqman Fast Advanced master mix (Thermo Fisher Scientific), 1 µl 20x Taqman primer/probe mix (Thermo Fisher Scientific), 4 µl nuclease-free water were prepared and subjected to real-time PCR with a QuantStudio7 instrument (Thermo Fisher Scientific). The thermal cycling conditions were 50°C for 2 minutes and 95°C for 20 seconds, followed by 40 cycles of 95°C for 1 second and 60°C for 20 seconds. Samples were run in triplicate and mean cycle threshold (Ct) values were applied to the standard curve generated in the same experiment to obtain corresponding copy number of bacteria then converted to copy number per mL. The qPCR assay has been qualified with lower limit of quantitation (LLOQ) and upper limit of quantitation (ULOQ) of 5 and 10^7 copies, respectively. For the limit of detection (LOD), a cycle threshold cut-off of 38 was set based on qualification experiments (Supplementary figure 2). The 16S rRNA gene copy number can vary from 5-6 copies per bacterium so measurements reflect total genome copies rather than an absolute measurement of bacterial load. All PCR data and LODs were normalized by blood or plasma volume input into the PCR reaction and represented graphically as copies of 16S gene per mL of clinical sample.

Supplemental Figures and Tables

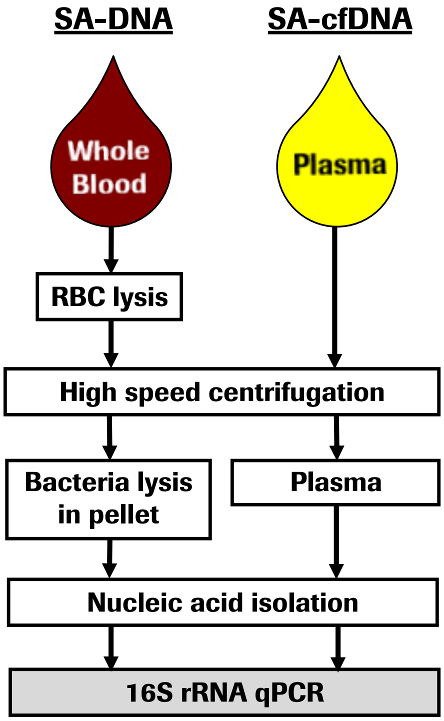

Supplementary Figure 1. Sample processing for SA-DNA and SA-cfDNA bacterial DNA quantifications.

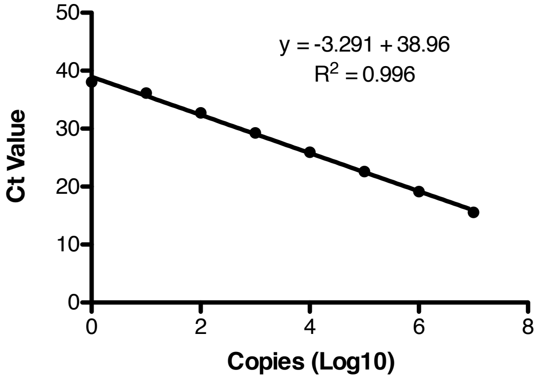

Supplementary Figure 2. Representative *S. aureus* genomic DNA standard curve.

**Pathogen**	**Source**	**Detected by qPCR**
Staphylococcus aureus USA300	ATCC® BAA-1556	+
Staphylococcus aureus USA300	NRS384	+
Staphylococcus aureus USA100	NRS382	+
Staphylococcus aureus USA400	NRS123	+
Staphylococcus aureus MN8	NRS112	+
Staphylococcus aureus Reynolds	NRS102	+
Staphylococcus aureus N315	NRS70	+
Staphylococcus aureus Mu50	NRS1	+
Staphylococcus aureus COL	NRS100	+
Staphylococcus aureus Newman	ATCC® 25904	+
Klebsiella pneumonia	ATCC® BAA-1705D-5	BLD
Acinetobacter baumannii	ATCC® 19606D-5	BLD
Enterococcus faecalis	ATCC® 29212Q-FZ	BLD
Salmonella enterica	ATCC® 700931D-5	BLD
Streptococcus pneumoniae	ATCC® 33400D-5	BLD
Pseudomonas aeruginosa	ATCC® 47085D-5	BLD
Bacteroides fragilis	ATCC® 25285D-5	BLD
Escherichia coli	ATCC® 10798D-5	BLD
Human genomic DNA	Promega	BLD

Below limit of detection (BLD)

Supplementary Table 1. DNA of a variety of organisms was tested for cross-reactivity with the *S. aureus* specific qPCR primers (>10 ng or >1 x 10^7 copies/reaction DNA for bacteria strains and 500ng/reaction for human genomic DNA).

Characteristic	Patient data (n = 73)
Age (Min-Max)	51 (21-90)
Male / Female	59 /14
MSSA / MRSA	48 / 25
In-hospital mortality	5
Treatment duration, mean days ±SD	42 ±23
Infection Source	
Line	6
Endocarditis	13
Skin & soft tissue infection	10
Pneumonia	5
Osteoarticular	32
Urinary tract infection	3
Unknown	4

Supplementary Table 2. Demographic and clinical data of bacteremia patient cohort.

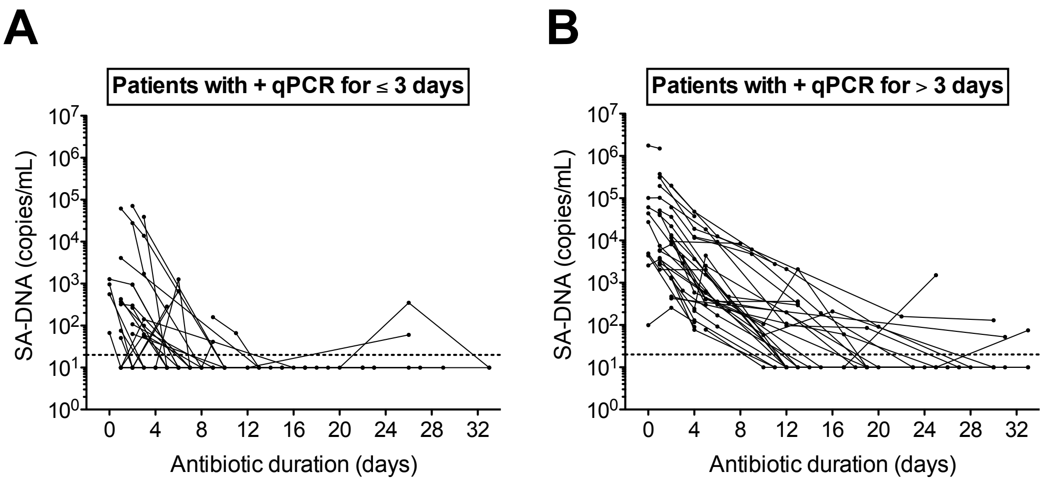

Supplementary Figure 3. *S. aureus* DNA levels in blood from longitudinal bacteremic patients separated based on duration of qPCR positive signal (A, B). Lines denote data from individual patients over time. Day 0 indicates start of empiric antibiotics. Dashed line: Limit of detection (20 copies/mL).

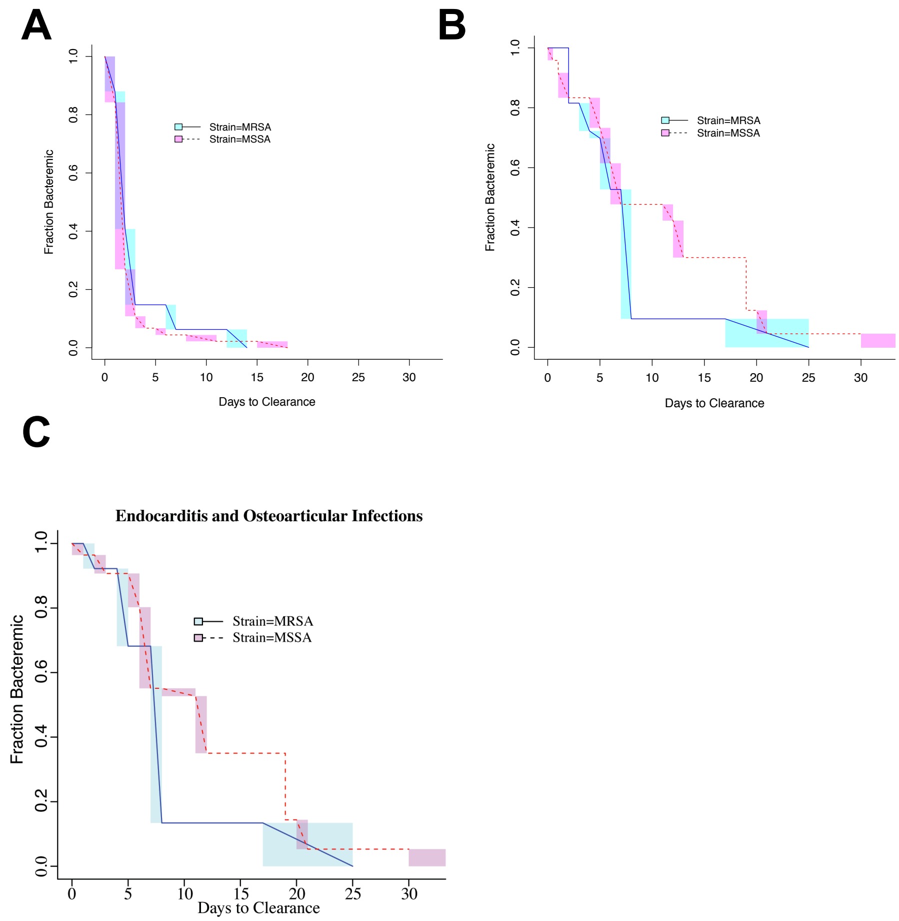

Supplementary Figure 4. Longer duration of SA-DNA positivity in patients with MSSA vs MRSA infections. A. Duration of blood culture positivity in patients with MRSA vs MSSA. B. Longer duration of SA-DNA detectable in blood from patients with MSSA infections vs. MRSA. C. Longer duration of SA-DNA detected in blood from patients with MSSA infections vs. MRSA in patients with complicated endocarditis or osteoarticular infections. Shading indicates the uncertainty in the interval censored data.

	Culture Time to Positivity			SA-DNA			SA-cfDNA		
	Spearman r		P value	Spearman r		P value	Spearman r		P value
**Culture time to +**	1		N/A	-0.449	***	0.0003	-0.280	ns	0.0725
**SA-DNA**	-0.449	***	0.0003	1.000		N/A	0.714	****	<0.0001
**SA-cfDNA**	-0.280	ns	0.0725	0.714	****	<0.0001	1.000		N/A
**Serum PCT**	-0.343	**	0.0032	0.613	****	<0.0001	0.375	*	0.0144
**WBC counts**	0.073	ns	0.5443	0.228	ns	0.0742	0.245	ns	0.1179
**% Neutrophils**	-0.217	ns	0.0821	0.561	****	<0.0001	0.526	***	0.0008
**Antibiotic Duration**	-0.262	*	0.0273	0.375	**	0.0029	0.293	ns	0.0596
**High WBC Duration**	0.039	ns	0.7591	0.198	ns	0.1465	0.383	*	0.0176
**Blood culture + Duration**	-0.059	ns	0.6305	0.392	**	0.0021	0.368	*	0.0211
**SA-DNA+Duration**	-0.259	*	0.0500	0.686	****	<0.0001	0.516	**	0.0025

Supplementary Table 3. Spearman correlations between markers of clinical severity, initial qPCR bacterial load and blood culture time to positivity. PCT, blood counts, and bacterial PCR measurements were made within 3 days of starting antibiotic therapy.
